# Synonymous Codon Ordering: A Subtle but Prevalent Strategy of Bacteria to Improve Translational Efficiency

**DOI:** 10.1371/journal.pone.0033547

**Published:** 2012-03-14

**Authors:** Zhu-Qing Shao, Yan-Mei Zhang, Xue-Ying Feng, Bin Wang, Jian-Qun Chen

**Affiliations:** State Key Laboratory of Pharmaceutical Biotechnology, School of Life Sciences, Nanjing University, Nanjing, Jiangsu Province, China; Columbia University, United States of America

## Abstract

**Background:**

In yeast coding sequences, once a particular codon has been used, subsequent occurrence of the same amino acid tends to use codons sharing the same tRNA. Such a phenomenon of co-tRNA codons pairing bias (CTCPB) is also found in some other eukaryotes but it is not known whether it occurs in prokaryotes.

**Methodology/Principal Findings:**

In this study, we focused on a total of 773 bacterial genomes to investigate their synonymous codon pairing preferences. After calculating the actual frequencies of synonymous codon pairs and comparing them with their expected values, we detected an obvious pairing bias towards identical codon pairs. This seems consistent with the previously reported CTCPB phenomenon, since identical codons are certainly read by the same tRNA. However, among co-tRNA but non-identical codon pairs, only 22 were often found overrepresented, suggesting that many co-tRNA codons actually do not preferentially pair together in prokaryotes. Therefore, the previously reported co-tRNA codons pairing rule needs to be more rigorously defined. The affinity differences between a tRNA anticodon and its readable codons should be taken into account. Moreover, both within-gene-shuffling tests and phylogenetic analyses support the idea that translational selection played an important role in shaping the observed synonymous codon pairing pattern in prokaryotes.

**Conclusions:**

Overall, a high level of synonymous codon pairing bias was detected in 73% investigated bacterial species, suggesting the synonymous codon ordering strategy has been prevalently adopted by prokaryotes to improve their translational efficiencies. The findings in this study also provide important clues to better understand the complex dynamics of translational process.

## Introduction

The mRNA-to-protein translation is such a complex and energy-consuming cellular activity that organisms have evolved multiple strategies to optimize this process [1]–[5]. One such strategy, codon usage bias, has been intensively studied [3], [6]–[15]. Due to the redundancy of the genetic code, most amino acids are encoded by two or more synonymous codons. These synonyms are often not used in equal frequencies within and among genomes [3], [6], [7]. Codons that can be most rapidly read by abundant cellular tRNA sets were often found to occur more frequently than predicted in highly-expressed genes [8]–[11]. The usage bias towards these favored ‘optimal’ codons has been suggested to improve translational efficiency and/or accuracy of essential genes in a wide range of organisms [12]–[14].

However, gene expression levels do not always correlate with the degree of codon usage bias. In *Escherichia coli*, a considerable number of genes with high expression levels were found to have low levels of codon usage bias [16]. On the other hand, some rare codons could be translated at a high rate, in spite of a low abundance of their cognate tRNAs [17], [18]. Studies on the artificial GFP gene and bacterial endogenous genes both indicated that there was no significant correlation between the protein expression level and codon usage bias [19], [20]. Taken together, these lines of evidence suggest that codon usage bias may not be the sole major strategy that organisms employed to optimize translation, and many other factors need to be further considered to elucidate the determinants of translation efficiency [21], [22].

Codon context is another important factor that may influence translational efficiency [23]. It has been found that adjacent codon pairs, which encode either the same or different amino acids, have biased occurrence frequencies within a genome [24], [25]. An experimental study confirmed that a *de novo* synthesized poliovirus coat protein gene with hundreds of underrepresented adjacent codon pairs led to a decreased rate of translation [26]. A recent study has investigated subsequent occurrences of synonymous codons in yeast. [27]. Among the nine amino acids (Ile, Ala, Gly, Pro, Thr, Val, Arg, Leu, and Ser) studied, pairs of co-tRNA codons occurred much more frequently than expected in the yeast genome. To explain this co-tRNA codons pairing bias (CTCPB) phenomenon, the authors proposed a tRNA recycling model. In such a model, tRNA used in a former codon position was speculated to remain associated with the ribosome and so can be reused more efficiently than a different tRNA. The reused tRNA will then bind the second codon as it did previously and in yeast this strategy can result in a 30% increase in translation speed [27]. Both adjacent codon pairing bias and CTCPB support the idea that codons in coding sequences are likely arranged in an organized way.

Being a newly-found phenomenon, CTCPB has not been surveyed in any prokaryotic genome yet. As two distinct domains of life, prokaryotes and eukaryotes have different tRNA compositions and decoding strategies [28]. For example, in many bacteria, a modified uridine (U) in the first anticodon position of tRNA^Ala^ (UGC) would guarantee it to recognize all four synonymous codons of the Ala; whereas in eukaryotes, at least two different tRNA^Ala^ genes are required to recognize all four Ala codons [29]. It is unknown whether differences like this in many other codon families would lead to different bias patterns of codon pairing in bacteria to those observed in eukaryotes. In this work, we investigated synonymous codon pairing patterns in 773 bacterial genomes for all 18 degenerate codon families, and provided evidence of organized synonymous codon orders in prokaryotes.

## Results

### The synonymous codon pairs in *E. coli* have biased occurrence frequency

To gain some initial understanding of the codon ordering pattern in bacteria, we first investigated the *E. coli* O157:H7 strain Sakai genome, which has a size of 5.5 Mb and 5,229 protein-coding genes. Within each gene, two subsequently-occurring synonymous codons, which could be separated by any number of non-synonymous codons, are viewed as a synonymous codon pair. An N-fold degenerate codon family would therefore have N^2^ different type of pairs. For three-fold to six-fold codon families (translated as Ala, Arg, Gly, Ile, Leu, Pro, Ser, Thr, and Val), the actual occurrence frequencies of all possible synonymous codon pairs were calculated and their deviations away from the expected values were expressed as the number of standard deviations (SD, see Materials and Methods, also refer to [27]). In the Table 1 (and for details in Table S1), the SD values obtained in each codon family were highly variable. For example, in Ala codon family, the values range from −8.99 to +15.56 SDs. Two clear patterns were observed. Firstly, identical codon pairs (e.g. GCC-GCC) occurred with significantly higher frequencies than expected, as almost all SD numbers on the diagonal lines within each family (Table 1) are bigger than 3 SD. The only exception to this observation was the CTC-CTC pair in the Leu codon family, which has real frequency deviated +2.88 SD from the expectation. This is in according with the previous finding in yeast [27]. Secondly, a majority of non-identical synonymous codon pairs were underrepresented (less than −3 SDs). Only a few non-identical codon pairs showed positive deviations from their expected frequencies and notably, many of these pairs of codons ended with a thymine (T) and an adenosine (A) or with a Guanine (G) and an adenosine (A): such as GCT-GCA pairs (the order can be overturned) in the Ala codon family, CCT-CCA pairs in the Pro codon family, ACT-ACA pairs in the Thr codon family, GTT-GTA pairs in the Val codon family, CTT-CTA pairs in the Leu4 codon family, GGG-GGA pairs in the Gly codon family, CGG-CGA, AGG-AGA pairs in the Arg codon family, and TTG-TTA pairs in the Leu2 codon family. This suggested that the *E. coli* coding sequences had a tendency to preferentially pair for their respective amino acids' A- and T-ending synonymous codons or A- and G-ending synonymous codons together, which is interesting. To test whether the A/T-ending codon pairing preferences detected in above-mentioned amino acids (Ala, Leu4, Pro, Thr, and Val) are caused by AT rich nucleotide compositional bias near the start or stop coding regions [30], we precluded 50 codons from each end of coding genes and conducted the analyses again. The obtained results (Table S2) revealed that the overall codon pairing pattern had almost no changes. The A- and T-ending codon paring preferences could be still detected in these amino acids.

**Table 1:** Standard deviations from expected for codon pairs in three to six-fold degenerate codon families in *E. coli*.

Ala	GCC	GCT	GCA	GCG	tRNA	copy
GCC	**12.68**	−2.95	−5.69	−4.63	Ala-GGC	2
GCT	−0.55	**9.91**	**3.29**	−8.99		
GCA	−5.89	**5.69**	**9.23**	−6.15	Ala-TGC	3
GCG	−6.19	−8.85	−4.67	**15.56**		

Gly	GGC	GGT	GGA	GGG	tRNA	copy
GGC	**9.87**	−0.78	−9.22	−5.56	Gly-GCC	4
GGT	−2.46	**9.44**	−3.74	−6.28		
GGA	−9.16	−4.20	**15.47**	**6.75**	Gly-TCC	1
GGG	−3.33	−8.41	**6.25**	**11.63**	Gly-CCC	1

Pro	CCC	CCT	CCA	CCG	tRNA	copy
CCC	**11.31**	**6.73**	−0.612	−9.00	Pro-GGG	1
CCT	**5.68**	**7.22**	**4.65**	−9.77		
CCA	0.25	**3.72**	**7.04**	−6.58	Pro-TGG	2
CCG	−9.10	−9.882	−6.77	**15.59**	Pro-CGG	1

Thr	ACC	ACT	ACA	ACG	tRNA	copy
ACC	**12.43**	−3.29	−13.23	−2.50	Thr-GGT	2
ACT	−2.42	**8.75**	**3.024**	−6.01		
ACA	−13.82	**3.35**	**19.51**	0.25	Thr-TGT	1
ACG	−2.65	−5.23	−0.37	**7.57**	Thr-CGT	1

Val	GTC	GTT	GTA	GTG	tRNA	copy
GTC	**3.80**	−1.02	−1.25	−1.23	Val-GAC	2
GTT	−0.38	**10.32**	**5.77**	−12.18		
GTA	−3.03	**6.03**	**4.66**	−5.76	Val-TAC	5
GTG	−0.61	−11.87	−6.90	**15.15**		

Ile	ATC	ATT	ATA	tRNA	copy
ATC	**14.39**	−6.77	−14.33	Ile-GAT	3
ATT	−6.29	**7.26**	−3.00		
ATA	−15.16	−2.04	**37.43**		

Arg4	CGA	CGC	CGT	CGG	tRNA	copy
CGA	**9.83**	−5.91	−5.98	**3.97**	Arg-TCG	3
CGC	−6.94	**11.37**	1.43	−4.35		
CGT	−5.86	1.97	**13.25**	−7.45	Arg-ACG	4
CGG	**4.08**	−4.69	−8.35	**14.65**	Arg-CCG	1

Arg2	AGA	AGG	tRNA	copy
AGA	**34.68**	**20.58**	**Arg-** **TCT**	8
AGG	**25.61**	**18.03**	Arg-CCT	1

Leu4	CTC	CTT	CTA	CTG	tRNA	copy
CTC	2.88	0.68	−0.81	0.26	Leu-GAG	1
CTT	2.19	**8.028**	**4.51**	−10.80		
CTA	−0.40	**4.998**	**5.26**	−10.30	Leu-TAG	1
CTG	−0.93	−10.38	−7.91	**23.52**	Leu-CAG	3

Leu2	TTA	TTG	tRNA	copy
TTA	**20.36**	**5.85**	Leu-TAA	1
TTG	**4.94**	**7.94**	Leu-CAA	1

Ser4	TCC	TCT	TCA	TCG	tRNA	copy
TCC	**5.80**	1.13	−5.71	−4.12	Ser-GGA	2
TCT	**5.15**	**13.25**	2.94	−3.73	Ser-AGA	
TCA	−4.07	2.01	**10.55**	−0.64	Ser-TGA	1
TCG	0.01	−3.38	−0.15	**8.28**	Ser-CGA	1

Ser2	AGC	AGT	tRNA	copy
AGC	**12.38**	−0.09	Ser-GCT	1
AGT	−0.81	**5.35**	Ser-ACT	

**NOTE:** The nine multi-fold degenerate codon families (encoding Ala, Gly, Pro, Thr, Val, Ile, Arg, Leu, and Ser) were analyzed. For each family, all present tRNA species, copy numbers were shown.

**Figure 1:**
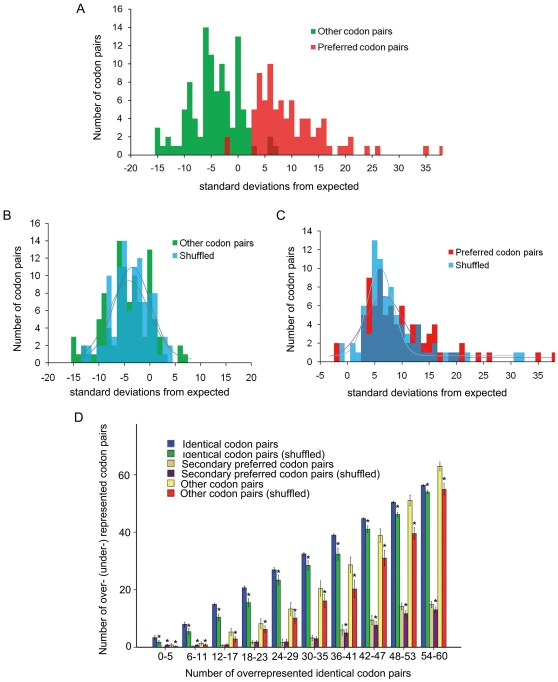
Within-gene-shuffling decreased the extents of synonymous codon pairing. A: SD values of preferred codon pairs (including identical and secondary preferred) and other codon pairs in *E. coli*. B: Comparisons of SD values for unpreferred codon pairs before and after shuffles. After shuffling SD values were increased (*P*<0.05) in *E. coli*. C: Comparisons of SD values for preferred codon pairs before and after shuffles. After shuffling SD values were significantly decreased (*P*<0.05) in *E. coli*. D: Comparisons of overrepresented identical codon pairs, secondary preferred codon pairs and underrepresented codon pairs before and after shuffles in 773 genomes. The frequencies of both overrepresented identical and secondary preferred codon pairs and underrepresented other codon pairs were significantly decreased in a majority of bacteria (* *P*<0.05).

It seems that the codon pairing pattern observed in *E. coli* genome is different from that reported in yeast and other eukaryotic genomes [27]. In yeast, codons that are recognized by the same tRNA were found to pair together preferentially. To investigate whether such a co-tRNA pattern is true in bacteria, we analyzed nine two-fold degenerate codon families (for Asn, Asp, Cys, Gln, Glu, His, Lys, Phe, and Tyr). For most of these families (except Gln), there is only one type of tRNA gene present in the *E. coli* genome (Table S3), and the tRNA could recognize both codons. In such a two-fold degenerate codon family, two codons can form four types of pairs (e.g. Phe codons would form UUU-UUU, UUU-UUC, UUC-UUU, and UUC-UUC pairs) and these four pairs would be expected to have similar occurrence frequencies, since they all meet the co-tRNA rule. However, the obtained data (Table S3) showed that it was not the case: identical codon pairs (e.g. UUU-UUU, UUC-UUC in the Phe codon family) often occurred at a much higher frequency than expected (deviated more than 3 SDs), while the frequencies of non-identical codon pairs (e.g. UUU-UUC, UUC-UUU in the Phe codon family) were significantly lower than expected (less than −3 SDs). When the results described in Tables 1 and S3 were analyzed together, we found that in *E. coli*, identical codon pairs are always favored in degenerate codon families (more than 3 SDs), while many non-identical codon pairs are not (Figure 1A).

### Within-gene-shuffling decreases the bias level of overrepresented synonymous codon pairs

To preclude a possibility that the observed biases of synonymous codon pairs in *E. coli* genome are due to an uneven distribution of different codons among different sets of genes (which may be caused by local variation of GC content), we performed the within-gene-shuffling to alter synonymous codon positions in every coding sequences, while maintaining their amino acid sequences and codon frequencies unchanged. As documented in a previous study [27], if selection force shapes the synonymous codon ordering and drives the preferred codon pairs to show up more frequently, within-gene-shuffling would disrupt such effects and the bias level of these pairs would decrease. In contrast, codon pairing bias caused by local GC content variation among different genes would not be affected by within-gene-shuffling.

Our data demonstrated that deviated SD values were significantly decreased for preferred codon pairs and increased for unpreferred codon pairs (*P*<0.001, based on 10000 times bootstrap samples) in *E. coli* (Figure 1B and 1C). It therefore suggests that the preferential codon pairs in *E. coli* are probably shaped by selection.

### The pattern of synonymous codon pairing is conserved in prokaryotes

**Figure 2:**
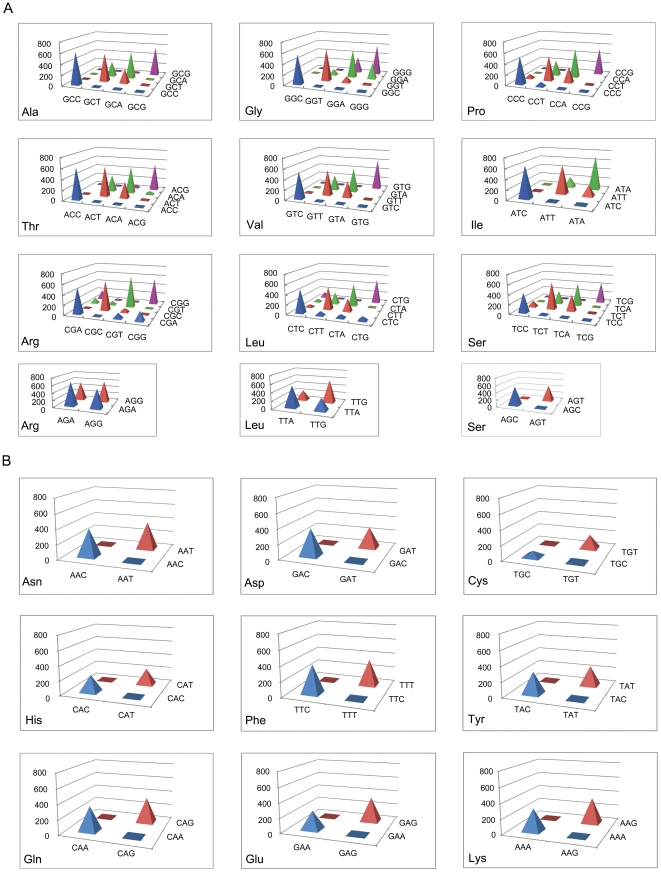
3D-pyramid distribution chart showing the variation of overrepresented codon pairs within different codon families. The vertical axis represents the total number of bacterial genomes in which a given synonymous codon pair is overused. A total of 773 bacterial genomes were analyzed. A: Distribution of overrepresented codon pairs in three-fold, four-fold, and six-fold degenerate codon families. The three examples of six-fold codon families were regarded as combined four-fold and two-fold codon families. B: The distribution of overrepresented codon pairs in two-fold degenerate codon families.

As analyzed above with the use of *E. coli* data, we described a synonymous codon pairing pattern that was different from that previously reported in eukaryotes [27]. To test whether this pattern is present universally in prokaryotes, we performed the same analysis in the genomes of 772 other bacteria. For each possible synonymous codon pair, we counted the total number of bacterial genomes in which the pair is overrepresented. As shown by Figure 2A and 2B, for all 18 amino acids, identical codon pairing is the most favored type of pairing among a total of 773 genomes surveyed, as the high peaks generally appeared along the diagonal lines in each codon family. However, there was also less marked overrepresentation of certain non-identical codon pairs in this genome-wide study as had previously been shown for *E.coli* (Figure 2A). These preferences included the T- and A-ending codon pairs in seven codon families (for Ala, Pro, Thr, Val, Ile, Leu4, and Ser4), and G- and A-ending codon pairs in four codon families (for Gly, Arg2, Arg4, and Leu2). Only a small number of other non-identical codon pairs were overrepresented in a limited number of bacterial genomes. For the two-fold degenerate codon families, almost no overrepresentation of non-identical codon pairs was observed among the 773 genomes (Figure 2B).

We also performed the within-gene-shuffling in the surveyed bacterial genomes. The shuffle also induced SD values to decrease significantly for preferred codon pairs, including all identical codon pairs and certain non-identical codon pairs (a total of 22 pairs mentioned above, referred to as secondary preferred codon pairs hereafter). Figure 1D also showed us that when all species are categorized into ten groups according to the total number of biased identical codon pairs (ranging from 0/59 to 59/59, see next section), the average numbers of overrepresented identical codon pairs, secondary preferred codon pairs and underrepresented other codon pairs were all significantly decreased in each group after performing the within-gene-shuffling.

### Variant levels of synonymous codon pairing bias among different bacterial species

The 18 degenerate codon families contain a total of 59 different codons, meaning that there are a maximum of 59 identical codon pairs to be overrepresented. Figure 2 demonstrates that not all 773 genomes would universally exhibit biased usage for a specific identical codon pair. To investigate the variant extents of identical codon pairing bias among different bacteria, we counted the total number of overrepresented identical codon pairs in each species. Redundant genomes for a same species were excluded and a total number of 510 genomes remained for this part of analysis.

**Figure 3:**
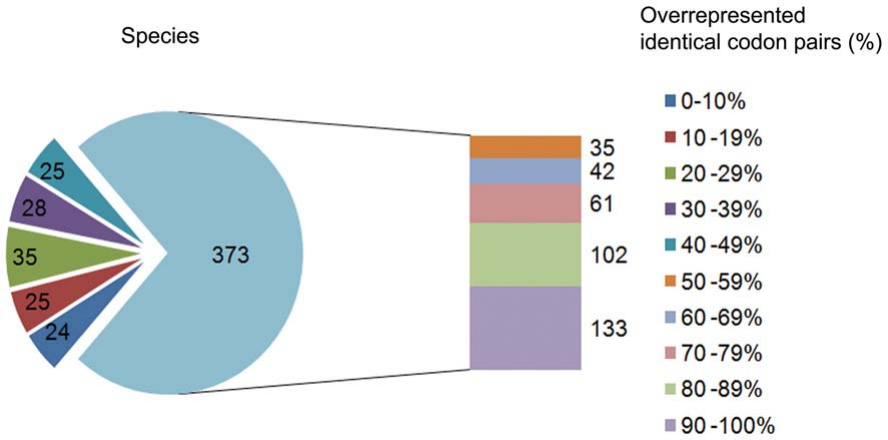
Variation of total overrepresented synonymous codon pairs among 510 bacterial species. Extents of variation of identical codon pairing in different bacteria; 373 species possessed more than 50% (30/59) overrepresented identical codon pairs. Among these, 296 species had >70% overrepresented identical codon pairs.

The total number of overrepresented identical codon pairs in surveyed genomes is strongly variable, ranged from 0 to 59 (out of 59). Notably, a majority of bacterial species have shown a high level of identical codon pairing bias. As shown in Figure 3, 73% of investigated bacterial species (373 out of 510) had more than 30 identical codon pairs (out of 59) overrepresented, including 296 species in which more than 42 identical codon pairs overused.

We then assessed the variant extents of identical codon pairing bias in different bacterial genera (Figure S1). Species belonging to a same genus often have similar numbers of total overused identical codon pairs, such as *Bordetella* that consists of 5 taxa, with overused identical codon pairs ranging from 49 to 57 (5 taxa, 49–57), *Corynebacterium* (7 taxa, 55–59), *Methylobacterium* (6 taxa, 48–55), *Pseudomonas* (7 taxa, 53–58), and *Shewanella* (12 taxa, 41–58). However, the extents of variation within certain genera could also be large, as shown in *Bacillus* (10 taxa, 18–47), *Bartonella* (5 taxa, 20–45), *Clostridium* (11 taxa, 24–47), *Lactobacillus* (14 taxa, 29–54), *Mycobacterium* (11 taxa, 16–57), *Mycoplasma* (12 taxa, 4–22), *Rickettsia* (11 taxa, 2–18), *Staphylococcus* (5 taxa, 15–31), *Streptococcus* (9 taxa, 23–44), and *Thermotoga* (5 taxa, 0–24). To gain some understanding on why species in a same genus would show such variation, two of these genera, *Mycobacterium* and *Rickettsia* were chosen for further investigation due to their relatively abundant information. Both genera contain 11 species in our data and the evolutionary states of these species have been well studied [31], [32].

**Figure 4:**
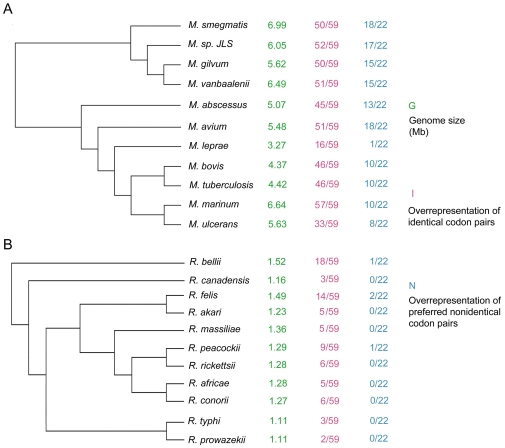
The proportion of overrepresented identical codon pairs can be variable even at genus level. Two phylogenetic trees of *Mycobacterium* and *Rickettsia* species were constructed separately, based on molecular data. For each species, its genome size, the total numbers of overused identical codon pairs and overused non-identical codon pairs in the secondary-favored group were shown. A: Genus *Mycobacterium* represents an example with overall high level of codon pairing pattern. The most reduced genome, found in *M. leprae*, has the lowest level of overrepresented codon pairs in the genus. B: Genus *Rickettsia* represents an example that has an overall low level of codon pairing. The early-diverging lineage of *R. bellii* has the highest level of overrepresented codon pairs, while other species in the genus, with reduced genomes to variable extents, also possess variant levels of overrepresented codon pairs.

Figure 4 shows the phylogenies of the two genera. For each species, its genome size and the total number of overused identical codon pairs were shown. In the genus *Mycobacterium* (Figure 4A), one distinctive species was *M. leprae*, which causes leprosy. It has a much reduced genome (3.27 Mb) in comparison to other species of the genus [31]. Interestingly, we found *M. leprae* had only 16 overrepresented identical codon pairs, while other species in the genus usually had more than 45. In the genus *Rickettsia* (Figure 4B), the early diverging lineage of *R. bellii* had the largest genomic size (1.52 Mb) among all surveyed species, as well as the highest number of preferred identical codon pairs (18/59). This was in comparison to some other species of the genus with reduced genomes, which usually had few overrepresented identical codon pairs, including *R. cannadensis* (1.16 Mb, 3/59), *R. typhi* (1.11 Mb, 3/59), and *R. prowazekii* (1.11 Mb, 2/59).

### Evolutionary conservation of secondary preferred non-identical codon pairs

The data from both *E. coli* (Table 1) and the summated bacterial data (Figure 2A) have shown that certain non-identical synonymous codon pairs are frequently overrepresented. For convenience, here we define the 59 identical codon pairs as the most-preferred group and the 22 often-preferred non-identical codon pairs as secondary preferred group: including the A- and T-ending codon pairs for Ala, Ile, Leu4, Pro, Ser4, Thr, and Val, and the A- and G-ending codon pairs for Arg2, Arg4, Gly and Leu2. We further investigated the correlation between the proportion of overused identical codon pairs and that of non-identical pairs. As shown in Figure S2, few non-identical codon pairs (mean<10%) were overused in species with a low percentage (<50%) of overrepresented identical codon pairs. As the proportion of preferred identical codon pairs further increases, the proportion of overused codon pairs belonging to the secondary preferred group also increased (Spearman ρ = 0.79, *P*<0.01). For other non-identical synonymous codon pairs that do not belong to the secondary preferred group, the value was maintained consistently at a low level.

In Figure 4, the final column shows the total number of overused non-identical codon pairs belonging to the secondary preferred group for all investigated *Mycobacterium* and *Rickettsia* species. As we mentioned above, overrepresentation of the secondary preferred group is positively correlated with identical codon pairing. In *Mycobacterium*, species with high levels (>45/59) of overused identical codon pairs also tend to have a high level (10–18 out of 22) of overused non-identical codon pairs. In the *Rickettsia* species, which possess lower levels (<50%) of overused identical codon pairs, a maximum of two out of 22 frequently-favored non-identical codon pairs were overrepresented. Interestingly, this was also true for *M. leprae*, the species with highly reduced genome in *Mycobacterium* genus.

## Discussion

For more than 30 years, codon usage bias has been intensively studied in a wide range of organisms and been regarded as an important strategy of organisms to optimize translational efficiency and/or accuracy [6]–[14]. Relatively little attention has been paid to other strategies. Recently, Cannarozzi and colleagues [27] investigated subsequently-occurring synonymous codon pairs and found an intriguing co-tRNA codon pairing pattern. A model of tRNA recycling was further proposed, and was supported from studies with regards to tRNA channeling [33]–[38]. However, this co-tRNA pairing pattern, only partially investigated in codons for nine of 18 amino acids, has not been surveyed in non-eukaryotic genomes. In this study, by systematically analyzing 773 bacterial genomes, we found that the synonymous codon pairing pattern in prokaryotes could not be fully explained by the previously reported co-tRNA rule in eukaryotes. Instead, a more elaborate rule is likely working to drive only some selected synonymous codons pairing together preferentially.

### A more subtle synonymous codon pairing pattern in bacteria

The results of the initial study in the *E. coli* genome and then on 772 other bacterial genomes revealed that overrepresented synonymous codon pairs are not randomly distributed in prokaryotes (Tables 1 and S3; Figure 2). The 59 identical codon pairs represented the primary overrepresented codon pairs, and a total number of 22 non-identical codon pairs formed the secondary overrepresented group. Other non-identical codon pairs, including those in two-fold degenerate codon families, were rarely found to be overrepresented.

**Figure 5:**
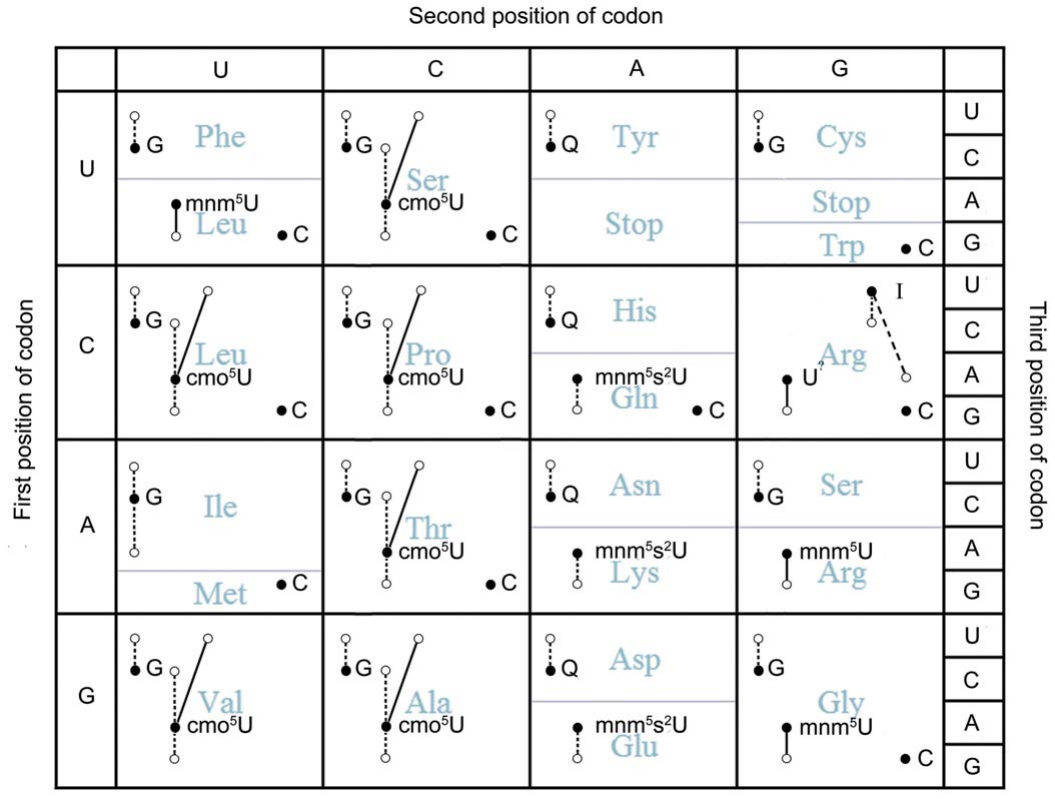
Different tRNA modifications are likely to correlate with the codon pairing patterns observed in bacteria. The location of a black dot represents the codon recognized by a cognate tRNA through Watson-Crick pairing, and the first anticodon base (with or without modifications) of the tRNA is provided next to the black dot. According to wobble rule, the tRNA is able to recognize other synonymous codons (white circles). However, our analysis of the synonymous codon pairing pattern in bacteria supports the hypothesis that the other synonymous codons are recognized in a discriminated way. In the Ala, Leu4, Pro, Ser4, Thr, and Val codon families, the tRNA with a modified cmo^5^U at the wobble position would prefer A- and U-ending codons (linked with solid lines), but avoid C- and G-ending codons (linked with dashed lines). However, in Arg2, Arg4, Gly, and Leu2 codon families the tRNA with a modified mnm^5^U (not confirmed for Arg4 as yet) at the wobble position would prefer A- and G-ending codons instead (linked with solid lines). In the Gln, Glu and Lys codon families, the tRNA with a modified mnm^5^S^2^U at the wobble base would only prefer A-ending codons and avoid G-ending codons (linked with dashed lines).

These results cannot be fully explained by the co-tRNA codon pairing pattern observed in yeast and other eukaryotic genomes [27]. Here we use the Ala codon family as an example to illustrate this point. In *E. coli* (as well as in many other bacterial genomes), there are two types of tRNA^Ala^ genes: tRNA^Ala^ (GGC) and tRNA^Ala^ (UGC). The guanine (G) wobble base in the former tRNA species would enable it to recognize both GCC and GCT codons, whereas the uridine (U) wobble base with the 5-carboxymethoxyuridine modification (cmo^5^U; Figure 5) in the latter tRNA species would allow the recognition of all four codons for Ala [29]. Taken together, it would be expected that the GCT/GCC codon pairs would be overrepresented according to the co-tRNA rule, since both types of tRNA can recognize them. However, only identical codon pairs and GCT/GCA codon pairs are frequently overrepresented for Ala in *E. coli* (and also other bacteria). Similar reasoning can also be done in other, especially four-fold, families based on Table 1. Therefore, we speculated that synonymous codon pairing pattern in bacteria is mainly biased towards identical codon pairs plus some selected non-identical codon pairs, rather than all co-tRNA pairs. These findings suggest that the codon pairing pattern in bacteria is more limited than the pattern reported in eukaryotes.

### Selection plays a role in shaping biased synonymous codon pairing in bacteria

There are two possible explanations for why a genome would have a biased order of synonymous codons: i) variation in the local GC content may cause different sets of genes to favor different synonymous codons; and ii) the evolutionary selection forces would shape the order of synonymous codons in a beneficial way. The first explanation has been thoroughly discussed and been largely excluded for eukaryotic data previously [27]. In this study, we also performed within-gene-shuffling of synonymous codons. After the shuffle, the bias level (expressed as SD values) was significantly decreased for preferred codon pairs and increased for other codon pairs in *E. coli* (Figure 1B, C). This is consistent with the results obtained in yeast, indicating selection force is shaping the synonymous codon orders in *E. coli* genes. Furthermore, if the local GC content variation indeed causes variant codon usages among different sets of genes, it would be expected that the G-/C-ending codon pairs are more likely to be overrepresented than expected. However, this was seldom the case in the 733 bacterial genomes studied herein, including many GC-rich genomes (Figure 2A). Finally, experiments on synthesized GFP genes have confirmed that favored synonymous codon pairing in yeast can greatly improve translational efficiency by up to 29% [27]. This strongly suggests that codon pairing bias is not simply the result of local GC content variation, but more likely shaped by translational selection. Based on these lines of evidence, we propose that the biased synonymous codon pairing pattern observed in prokaryotes would also have an effect to improve translational efficiency. The final decisive evidence would come from an elegantly designed experimental study.

### Hypotheses to explain the observed synonymous codon paring pattern in bacteria

Our observation in prokaryotes revealed that many co-tRNA codon pairs were actually not enriched. A reasonable explanation comes from the tRNA wobbling rule is that a given tRNA species would recognize multiple codons in a discriminating way, and only selected non-identical codons are preferred. Firstly, identical codon pairing would be indubitably recognized by a same tRNA with a high efficiency. This could explain why identical codon pairs in all 18 degenerate codon families are primarily overrepresented in bacteria. Secondly, codons that have high level of affinity/efficiency in interacting with a tRNA anticodon could be preferred over other recognizable codons with low affinity/efficiency.

In this study, a total number of 22 non-identical codon pairs were often found to be overrepresented in bacteria. To explore why only these pairs are preferred, we took variant modification ways of tRNA wobble base into consideration. In *E. coli*, very few codons are translated by tRNAs that underwent no modifications [39]. Over the last 40 years, many different modification modes of the tRNA wobble bases have been discovered [40]–[46]. Interestingly, one common modification on the U wobble base, namely cmo^5^U, was found on tRNA species in the Ala, Leu4, Pro, Ser4, Thr, and Val codon families [47] (Figure 5). This modification would enable the corresponding tRNA to recognize U-, A-, G- and C-ending codons on mRNA. A recent study found, contrary to the author's expectation, that tRNAs with wobble-U base (e.g., tRNA^Ala^ [UGC]) showed a high affinity level with A- and U-ending codons, but a low affinity with G- and C- ending codons [48]. The six families with cmo^5^U modifications also happen to be those exhibiting overrepresentation of the A- and T-ending codon pairs in our study (Figure 2A). These could explain why A- and T-ending codon pairs in these families were overrepresented in bacteria. Additionally, in Arg2, Gly and Leu2 codon families, the U wobble base of tRNA species underwent a different modification, called 5-methylaminomethyluridine (mnm^5^U) [49]. This enables the corresponding tRNA to recognize A- and G-ending codons only. Interestingly, these three families have been shown in our study to have an overrepresentation of A- and G-ending synonymous codon pairs (Figures 2 and 5). In two-fold degenerate codon families, such as for Gln, Glu and Lys, the tRNA species also have U wobble base but underwent another specific modification called 5-methylaminomethyl-2-thiouridine (mnm^5^s^2^U, Figure 5, [50]); no evidence of overrepresentation of A- and G-ending codon pairs has been found in these three two-fold degenerate codon families in bacteria. We speculate that the additional 2-thiouridine modification on wobble U may enable the tRNA to read A- and G-ending codons discriminately in these codon families. Indeed, previous study have shown that A-ending codon for Glu was read three times faster than the G-ending codon [51].

These observed correlations between preferred non-identical codon pairs and various tRNA modification ways are unlikely to be coincidental in our view. We speculate that different modifications of the tRNA wobble bases would modulate not only the specificity, but also the affinity/efficiency of tRNA molecules in recognizing different codons, which would further affect the synonymous codon pairing patterns in bacteria. Only identical codon pairs and non-identical codon pairs, in which two codons are recognized with equally (or closely) high affinity/efficiency by a same modified tRNA, would be favored by translational selection and accumulated in bacterial genomes.

### Conclusions

In this study, we investigated 773 bacterial genomes and found an interesting pattern of non-random usage of synonymous codon pairs. Identical codon pairs, as well as certain non-identical codon pairs, were overrepresented with significantly higher frequencies than expected in a majority of bacterial species, suggesting a universal need for improving translational efficiency during the evolution of prokaryotes. Different modifications on tRNA wobble bases were found to have a good correlation with the identified non-identical codon paring pattern. We conclude that prokaryotes adopted a subtle but prevalent codon ordering strategy to optimize their translational efficiencies.

## Materials and Methods

### Databases

Protein coding sequences (CDS) for all 773 bacterial genomes were retrieved from the NCBI ftp server (ftp://ftp.ncbi.nih.gov/genomes). The list of all genomes was provided in Table S4.

### Calculation of synonymous codon pair frequencies and their deviations in E. coli

In *E. coli* O157:H7 strain Sakai (GenBank accession number: Nc_002695), the actual occurrence number and frequency of each synonymous codon pair in every degenerate codon family were first calculated. The expected frequency of each codon pair was computed as the products of the frequencies of two individual codons in the genome. To quantify the extents of deviation away from the expected value, the method used by Cannarozzi et al. [27] was adopted: the expected number was subtracted from the observed number and divided by the standard deviation (estimated assuming a binomial distribution). Synonymous codon pairs with an actual occurrence frequency that deviated away from the expected value by more than three SDs, negatively or positively, were regarded as under- or over-represented codon pairs, respectively.

### Investigating overrepresented synonymous codon pairs in 772 bacterial genomes

For each of the other 772 bacterial genomes, as done in *E. coli*, all overrepresented synonymous codon pairs (>3 SDs) were obtained. For all possible types of synonymous codon pairs, the total numbers of bacterial genomes showing overrepresentation were then summated. The obtained data were used to draw a serial of 3D-pyramid distribution charts, separated into individual families.

Eighteen degenerate codon families have a total of 59 identical codon pairs. To investigate the variant extents of deviated codon pairs in different bacteria, the total numbers of overrepresented identical codon pairs in 510 bacterial species (redundant genomes for a same species were excluded) were calculated and categorized. Besides the 59 identical codon pairs, some non-identical codon pairs (a total number of 22 pairs: GCT/A-GCA/T, ATT/A-ATA/T, CCT/A-CCA/T, ACT/A-ACA/T, GTT/A-GTA/A, CTT/A-CTA/T, TCT/A-TCA/T, CGA/G-CGG/A, AGA/G-AGG/A, GGA/G-GGG/A and TTA/G-TTG/A) were also found to be frequently overrepresented in bacterial genomes. The correlation between the occurrences of these preferred non-identical and identical codon pairs among different bacterial species were analyzed with Spearman's correlation test.

### Within-gene-shuffling of synonymous codon

Maintaining the order and content of amino acids unchanged, the synonymous codons were shuffled within each gene in *E. coli* genome and other 772 bacterial genomes. The SD values of each codon pair after the shuffles were then compared with the observed data. 10000 times bootstrap was performed to get a *P* value less than 0.001. In *E. coli*, the distribution of SD values of preferred codon pairs and other non-preferred codon pairs before and after the shuffles were drawn. For all 773 genomes, the variation in total numbers of identified overrepresented preferred codon pairs and underrepresented other codon pairs before and after the shuffles was also analyzed. For the ten groups divided based on overall overrepresented identical codon pairs, the significance of identical codon pair changes for each group was tested by using paired-t-test.

### Drawing phylogenetic trees for genera *Mycobacterium* and *Rickettsia*

Two representative genera, *Mycobacterium* and *Rickettsia*, were chosen to investigate the extent of variability in total number of overused identical codon pairs among close-related species, as well as to explore possible explanations. 5S-23S-16S rDNA sequences of each genome were extracted to build the *Mycobacterium* tree. The tree containing 11 species was constructed by using MEGA 4 with Maximum-Likelihood method [52]. The tree of 11 *Rickettsia* species was constructed by concatenating *atp*A, *glt*A, and 16S rDNA sequences as described previously [31]. For all species in the two genera, their genome sizes, the total numbers of overused identical codon pairs, and the total numbers of overused non-identical codon pairs belonging to the preferred group were compared.

## Supporting Information

Figure S1 **Variant extents of total overrepresented identical codon pairs in bacteria.** The phylogeny of the 510 bacterial species was built using the online server of iTOL (interactive Tree of Life: http://itol.embl.de/). Total numbers of overrepresented identical codon pairs in all bacterial genome are labeled.
(PDF)

Figure S2 **The proportion of overrepresented non-identical codon pairs in the secondary-preferred group (22 pairs) is positively correlated to that of overrepresented identical codon pairs (59 pairs).**
(PDF)

Table S1 **Co-occurrence counts, expected value, percent and standard deviations from expected for pairs of three to six-fold degenerate codon families** ***in E. coli*** **.**
(XLS)

Table S2 **Standard deviations from expected for codon pairs in three-fold to six-fold codon families in** ***E. coli*** **(excluding first and last 50 codons of each gene).**
(DOC)

Table S3 **Standard deviations from expected for codon pairs in two-fold degenerate codon families in** ***E. coli*** **.**
(DOC)

Table S4 **773 bacterial genomes analyzed in this study.**
(XLS)
