# Supplementary figures and images for: Synonymous Codon Ordering: A Subtle but Prevalent Strategy of Bacteria to Improve Translational Efficiency
Source: PLoS One. 2012 Mar 14;7(3):e33547. doi: 10.1371/journal.pone.0033547 (PMC3303843; doi:10.1371/journal.pone.0033547)

I

S

I Overrepresented identical codon pairs

S Species

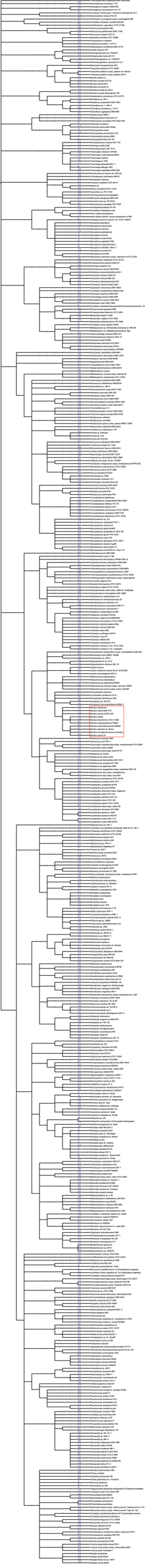

Supplement: Figure S1 — Variant extents of total overrepresented identical codon pairs in bacteria. The phylogeny of the 510 bacterial species was built using the online server of iTOL (interactive Tree of Life: http://itol.embl.de/). Total numbers of overrepresented identical codon pairs in all bacterial genome are labeled. (PDF) [file pone.0033547.s001.pdf]

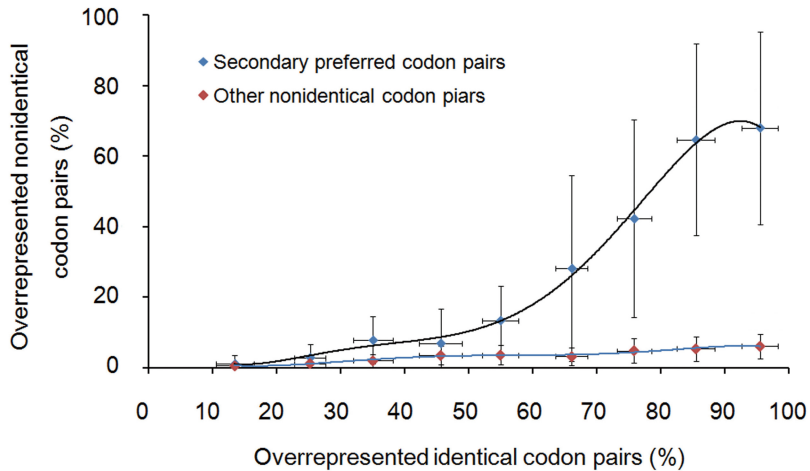

Supplement: Figure S2 — The proportion of overrepresented non-identical codon pairs in the secondary-preferred group (22 pairs) is positively correlated to that of overrepresented identical codon pairs (59 pairs). (PDF) [file pone.0033547.s002.pdf]
