# Supplementary material for: Synonymous Codon Ordering: A Subtle but Prevalent Strategy of Bacteria to Improve Translational Efficiency
Source: PLoS One. 2012 Mar 14;7(3):e33547. doi: 10.1371/journal.pone.0033547 (PMC3303843; doi:10.1371/journal.pone.0033547)
Supplement: Table S2 — Standard deviations from expected for codon pairs in three-fold to six-fold codon families in E. coli (excluding first and last 50 codons of each gene). (DOC) [file pone.0033547.s004.doc]

**Table S2: Standard deviations from expected for codon pairs in E. coli (excluding the first and last 50 codons of each gene).**

| **Ala** | GCC | GCT | GCA | GCG | tRNA | Copy |
| --- | --- | --- | --- | --- | --- | --- |
| GCC | **13.35** | -3.42 | -3.96 | -6.29 | Ala-GGC | 2 |
| GCT | 0.76 | **11.08** | **3.44** | -10.89 |  |  |
| GCA | -6.90 | **5.75** | **9.32** | -5.23 | Ala-TGC | 3 |
| GCG | -6.86 | -9.08 | -6.26 | **17.40** |  |  |
|  | | | | | | |
| **Gly** | GGC | GGT | GGA | GGG | tRNA | copy |
| GGC | **12.07** | -1.19 | -11.05 | -6.75 | Gly-GCC | 4 |
| GGT | -2.42 | **9.62** | -2.83 | -7.48 |  |  |
| GGA | -12.56 | -3.96 | **17.24** | **10.32** | Gly-TCC | 1 |
| GGG | -3.81 | -8.29 | **6.43** | **12.18** | Gly-CCC | 1 |
|  | | | | | | |
| **Pro** | CCC | CCT | CCA | CCG | tRNA | copy |
| CCC | **11.78** | **6.86** | -1.02 | -8.65 | Pro-GGG | 1 |
| CCT | **3.68** | **5.80** | **5.35** | -8.04 |  |  |
| CCA | -0.20 | **4.31** | **9.13** | -7.65 | Pro-TGG | 2 |
| CCG | -7.53 | -9.04 | -7.86 | **14.54** | Pro-CGG | 1 |
|  | | | | | | |
| **Thr** | ACC | ACT | ACA | ACG | tRNA | copy |
| ACC | **11.14** | -0.81 | -12.63 | -3.29 | Thr-GGT | 2 |
| ACT | -1.46 | **7.08** | 2.04 | -5.09 |  |  |
| ACA | -12.91 | **3.66** | **19.50** | -1.129 | Thr-TGT | 1 |
| ACG | -2.67 | -7.04 | -0.13 | **8.70** | Thr-CGT | 1 |
|  | | | | | | |
| **Val** | GTC | GTT | GTA | GTG | tRNA | copy |
| GTC | 2.32 | -2.67 | -0.80 | 0.95 | Val-GAC | 2 |
| GTT | -0.26 | **13.20** | **6.04** | -14.48 |  |  |
| GTA | -1.28 | **5.90** | **4.06** | -6.41 | Val-TAC | 5 |
| GTG | -0.70 | -12.66 | -6.92 | **15.55** |  |  |
|  | | | | | | |
| **Ile** | ATC | ATT | ATA |  | tRNA | copy |
| ATC | **14.98** | -8.26 | -13.36 |  | Ile-GAT | 3 |
| ATT | -7.76 | **8.44** | -2.41 |  |  |  |
| ATA | -14.47 | -1.32 | **36.44** |  |  |  |

**Table S2. Continued**

| **Arg4** | CGA | CGC | CGT | CGG | tRNA | copy |
| --- | --- | --- | --- | --- | --- | --- |
| CGA | **8.76** | -6.04 | -2.98 | **3.34** | Arg-TCG | 3 |
| CGC | -5.16 | **10.97** | 0.14 | -5.49 |  |  |
| CGT | -3.97 | 1.09 | **13.31** | -8.44 | Arg-ACG | 4 |
| CGG | 1.67 | -5.38 | -8.82 | **18.66** | Arg-CCG | 1 |
|  | | | | |  | |
| **Arg2** | AGA | AGG | tRNA | copy |  | |
| AGA | **40.17** | **20.59** | **Arg-TCT** | 8 |  | |
| AGG | **22.44** | **17.35** | Arg-CCT | 1 |  | |
|  | | | | |  | |
| **Leu4** | CTC | CTT | CTA | CTG | tRNA | copy |
| CTC | 1.33 | -0.82 | -2.55 | 1.23 | Leu-GAG | 1 |
| CTT | 1.93 | **6.742** | **4.03** | -8.94 |  |  |
| CTA | 0.69 | **7.042** | **5.57** | -11.69 | Leu-TAG | 1 |
| CTG | 0.10 | -8.652 | -7.84 | **20.98** | Leu-CAG | 3 |
|  | | | | |  | |
| **Leu2** | TTA | TTG | tRNA | copy |  | |
| TTA | **19.69** | **6.14** | Leu-TAA | 1 |  | |
| TTG | **4.66** | **9.50** | Leu-CAA | 1 |  | |
|  | | | | |  | |
| **Ser4** | TCC | TCT | TCA | TCG | tRNA | copy |
| TCC | **7.27** | 1.28 | -4.93 | -2.65 | Ser-GGA | 2 |
| TCT | **7.08** | **14.33** | 2.34 | -4.47 |  |  |
| TCA | -5.29 | -0.23 | **10.78** | 0.83 | Ser-TGA | 1 |
| TCG | 1.80 | -2.76 | -2.13 | **8.47** | Ser-CGA | 1 |
|  |  |  |  |  |  | |
| **Ser2** | AGC | AGT | tRNA | copy |  | |
| AGC | **12.60** | 0.81 | Ser-GCT | 1 |  | |
| AGT | 0.18 | **6.64** |  |  |  | |

**NOTE:** Three SD values inconsistent with table 1 are shown in shadow.
