# Supplementary material for: Synonymous Codon Ordering: A Subtle but Prevalent Strategy of Bacteria to Improve Translational Efficiency
Source: PLoS One. 2012 Mar 14;7(3):e33547. doi: 10.1371/journal.pone.0033547 (PMC3303843; doi:10.1371/journal.pone.0033547)
Supplement: Table S3 — Standard deviations from expected for codon pairs in two-fold degenerate codon families in E. coli . (DOC) [file pone.0033547.s005.doc]

**Table S3: Standard deviations from expected for different codon pairs in two-fold degenerate codon families in *E. coli*.**

| **Asn** | AAC | AAT | tRNA | copy |
| --- | --- | --- | --- | --- |
| AAC | **14.55** | -15.28 | Asn-GTT | 4 |
| AAT | -15.23 | **16.07** | Asn-ATT |  |
|  |  |  |  |  |
| **Asp** | GAC | GAT | tRNA | copy |
| GAC | **6.49** | -5.29 | Asp-GTC | 3 |
| GAT | -5.28 | **4.57** | Asp-ATC |  |
|  |  |  |  |  |
| **Cys** | TGC | TGT | tRNA | copy |
| TGC | **3.41** | -3.64 | Cys-GCA | 1 |
| TGT | -3.651 | **3.94** | Cys-ACA |  |
|  |  |  |  |  |
| **His** | CAC | CAT | tRNA | copy |
| CAC | **7.12** | -6.37 | His-GTG | 1 |
| CAT | -6.38 | **5.84** | His-ATG |  |
|  |  |  |  |  |
| **Phe** | TTC | TTT | tRNA | copy |
| TTC | **11.86** | -10.62 | Phe-GAA | 2 |
| TTT | -10.50 | **9.61** | Phe-AAA |  |
|  |  |  |  |  |
| **Tyr** | TAC | TAT | tRNA | copy |
| TAC | **7.19** | -6.51 | Tyr-GTA | 3 |
| TAT | -6.48 | **5.98** | Tyr-ATA |  |
|  |  |  |  |  |
| **Gln** | CAA | CAG | tRNA | copy |
| CAA | **13.00** | -9.70 | Gln-TTG | 2 |
| CAG | -9.76 | **8.09** | Gln-CTG | 2 |
|  |  |  |  |  |
| **Glu** | GAA | GAG | tRNA | copy |
| GAA | 2.411 | -2.90 | Glu-TTC | 2 |
| GAG | -2.92 | **3.92** | Glu-CTC |  |
|  |  |  |  |  |
| **Lys** | AAA | AAG | tRNA | copy |
| AAA | 2.60 | -3.30 | Lys-TTT |  |
| AAG | -3.32 | **5.43** | Lys-CTT | 5 |

**NOTE:** In each family, the present tRNA species, copy number were shown.
